# Progress of Interference of Traditional Chinese Medicine on Cirrhosis Treated with Bone Marrow Mesenchymal Stem Cells

**DOI:** 10.1155/2021/5569274

**Published:** 2021-05-11

**Authors:** Yaxin Wang, Huicun Zhang, Hongbing Wang

**Affiliations:** ^1^Beijing Hospital of Traditional Chinese Medicine, Capital Medical University, Beijing 100010, China; ^2^Beijing Institute of Traditional Chinese Medicine, Beijing 100010, China; ^3^Beijing Hospital of Traditional Chinese Medicine Yanqing Hospital, Beijing 102199, China

## Abstract

Transplantation of bone marrow mesenchymal stem cells has attracted more and more attention as a regenerative therapy for the treatment of liver diseases. A large number of studies have shown that this kind of cells can inhibit the activation of hepatic stellate cells and regulate tissue homeostasis and immune system via a variety of ways. Meanwhile, bone marrow mesenchymal stem cells can inhibit apoptosis of hepatocyte, improve liver function, and reduce inflammation through multiple pathways. These cells have a broad prospect in the treatment of liver cirrhosis. At present, there are many studies on the specific mechanism of bone marrow mesenchymal stem cells transplantation in the treatment of liver cirrhosis. This paper reviews the pathogenesis of liver cirrhosis and the mechanism of bone marrow mesenchymal stem cells transplantation in the treatment of liver cirrhosis, discusses the effectiveness of traditional Chinese medicine method in enhancing the efficacy of bone marrow mesenchymal stem cells transplantation, and looks forward to its application prospect in the future.

## 1. Introduction

Liver cirrhosis is the end stage of chronic liver disease. The progress of chronic liver disease can cause a variety of body damage, such as upper gastrointestinal bleeding, infection, hepatic encephalopathy, hepatocellular carcinoma, hepatorenal syndrome, and other malignant diseases [1]. Liver cirrhosis, contributing to more than 1 million deaths annually, is a major healthcare concern worldwide [2]. Patients with end-stage liver cirrhosis often require liver transplantation. But this treatment is faced with many problems that are difficult to solve, for example, the shortage of liver source, high immune rejection, being prone to postoperative complications [3], and high cost associated with the operation, so its clinical application is limited [4]. In order to prevent liver disease from developing into its end-stage, mesenchymal stem cell (MSC) therapy has been adopted and has shown its potential in curing this kind of liver disease. As an individualized cell therapy, MSCs transplantation has been deeply studied and has a broad prospect of clinical application. Bone marrow mesenchymal stem cells (BMSCs), of which the genetic background is relatively stable and the use rarely involves ethical problems, can be easily isolated and greatly expanded in vitro. By using these cells, the implantation reaction in vivo is weak, the rejection of transplantation can be avoided, and its allogeneic cell transplantation does not pose a risk of xenovirus infection. It has attracted much attention on how to improve the survival rate, increase proliferation ability of transplanted BMSCs, induce it to differentiate into mature hepatocytes, change the damaged liver microenvironment, and make it safe for clinical use. This paper reviews the mechanism of BMSCs transplantation in the treatment of liver cirrhosis and looks forward to its application prospect in the treatment of liver disease.

### 1.1. MSCs: Biology and Origins

In 1976, Friedenstein et al. [5] first expanded stromal cells obtained from marrow cell suspensions in vitro and identified discrete colonies of plastic-adherent. Each colony was named a colony-forming unit fibroblast (CFU-F). Cells that produced CFU-F colonies were named osteogenic stem cells or bone marrow stromal stem cells [6]. These cells exhibited a huge in vitro expansion and a potential to differentiate into adipocytes, chondrocytes, and osteocytes. The isolation of MSCs according to current criteria produces heterogeneous, non-clonal cultures of stromal cells containing stem cells with different multipotential properties, committed progenitors, and differentiated cells. To improve the characterization of MSCs, the International Society for Cellular Therapy (ISCT) proposed three rules to identify MSCs. According to the ISCT, MSCs must be purified from the bone marrow stromal population based on plastic adherence under standard culture conditions; MSCs must express the CD105, CD90, and CD73 antigens. Moreover, they should be CD45 (−) and CD34 (−). In addition, MSCs must differentiate in vitro into osteocytes, chondrocytes, and adipocytes [7]. The characteristics of MSCs above make them play an important role in clinic and experiment. Now most of the MSCs used in the study of liver cirrhosis are isolated from bone marrow. Using BMSCs in the treatment of liver cirrhosis has the following advantages: (1) they have a wide range of sources and are convenient to obtain; (2) they have a high ability of self-renewal and can be expanded massively in vitro; (3) they can differentiate into a variety of tissue types under the induction of appropriate culture medium and factors; (4) the multidirectional differentiation characteristics of MSCs will not be affected after being transferred into exogenous genes; (5) there are no problems such as tissue matching and immune rejection. The study of the mechanism of BMSCs in the treatment of liver cirrhosis is helpful to solve the existing problems.

### 1.2. Pathogenesis of Liver Cirrhosis

Liver cirrhosis is the final pathological result of various chronic liver diseases, and fibrosis is the precursor of cirrhosis. According to the existing research, the central link of liver cirrhosis is the activation of hepatic stellate cells (HSCs). When multiple molecular signal pathways in HSCs are activated, the molecular biological characteristics of hepatic stellate cells can be changed at the gene level, which can induce liver fibrosis. HSCs reside in the space of Disse in the normal liver and their main function is storage of Vitamin A and other retinoids [8]. Normally, HSCs are in a state of rest; when the liver is injured by inflammation or mechanical stimulation, pro-inflammatory factors such as platelet-derived growth factor (PDGF), tumour necrosis factor- (TNF-) *α*, transforming growth factor- (TGF-) *β*, and interleukin- (IL-) 1 can activate HSCs. After HSCs are activated, they will lose the oil droplets storing Vitamin A, proliferate under the stimulation of growth factors, and differentiate into myofibroblasts. Activated HSCs move to the injured site induced by monocyte chemoattractant protein-1 (MCP-1) and chemokine ligand 2 (CXCR2) and synthesize a large amount of extracellular matrix (ECM) and fibrotic collagen (I, II). The activity of matrix metalloproteinases (MMPs) will be inhibited and the degradation of ECM will be reduced by the overexpression of tissue matrix metalloproteinase inhibitors (TIMPs) [9]. The synthesis of ECM, which is mainly composed of collagen, was increased, while the degradation was relatively insufficient, and the total collagen could be increased to 3–10 times of the normal. Too much collagen deposited in the Disse space, resulting in the widening of the gap and the formation of the basement membrane under the hepatic sinusoid endothelial cells (LSECs). The reduction or even disappearance of the superior window pore of the endothelial cells would form a diffuse barrier and a capillarization of the hepatic sinusoid [10]. With the flattening of villi on the surface of hepatocytes and the formation of barriers, the transport of substances in hepatic sinusoids through the walls of hepatic sinusoids to hepatocytes was blocked, which interfered with the function of hepatocytes directly, resulting in the dysfunction of synthesis of hepatocytes. The stenosis of hepatic sinusoid, obstruction of blood flow, and increase of intrahepatic resistance affect the hemodynamic of portal vein, cause hypoxia of hepatocytes and disturbance of nutrient supply, aggravate hepatocyte necrosis, and make initiator factors work continuously. The fibre bundle of the portal area and hepatic capsule extend to the central vein of the hepatic lobule. These fibre septa encircle the regenerated nodules or re-segment the residual hepatic lobules and transform them into pseudo-lobules, forming a typical histopathological morphology of liver cirrhosis.

## 2. Mechanism of BMSCs in Liver Cirrhosis

Transplantation of BMSCs into damaged liver to reconstruct liver structure and improve liver function is of great significance for the treatment of liver fibrosis and liver cirrhosis. The mechanism of BMSCs in the treatment of liver cirrhosis is reviewed below. It is pointed out that there are still some outstanding problems of BMSCs transplantation in the treatment of liver cirrhosis.

### 2.1. Inhibiting the Activation of Hepatic Stellate Cells

The activation of HSCs is an important factor in hepatic fibrosis. The main function is to synthesize ECM components in normal liver tissue or liver cirrhosis. We know that liver cirrhosis is the repair response of the liver to various acute and chronic stimulation injuries. In the process of liver injury, HSC is an important cell for hepatocytes to produce ECM. BMSCs can inhibit the proliferation and activation of HSCs. Sakaida [11] confirmed that portal vein injection of BMSCs can inhibit the activity of HSCs and the expression of inflammatory molecules, protect the function of mitochondria, reduce oxidative stress, and thus improve the regeneration of rat hepatocytes and reduce the fibrosis of regenerated liver. Previous studies have shown that growth factors and cytokines secreted by BMSCs can inactivate HSCs and inhibit the progress of liver fibrosis. In in vitro experiments, the co-culture of BMSCs and HSCs can lead to cell cycle arrest in G10/G1 phase and cell apoptosis and ultimately inhibit the proliferation of HSCs [12]. Cao et al. [13] found that BMSCs can inhibit the expression of actin alpha 2 (ACTA2) and hepatocyte growth factor (HGF), initiate paracrine action, secrete a variety of anti-fibrosis cytokines and growth factors, induce HSCs apoptosis, reduce the deposition of ECM, and inhibit the formation of liver inflammation and fibrosis.

### 2.2. Participating in Immune Regulation

Liver fibrosis is the inevitable result of liver damage and chronic inflammation, and it is the necessary stage for the development of liver cirrhosis. Any cause of liver inflammation is characterized by inflammatory cell infiltration. The immunoregulatory properties of BMSCs are facilitated by their interactions with immune cells like T cells, B cells, dendritic cells, macrophages, and natural killer (NK) cells in a context and microenvironment-dependent manner [14]. The pro-fibrotic factors secreted by these cells play an important role in the activation and proliferation of HSCs [15]. These cells interact with liver parenchymal cells or nonparenchymal cells and jointly participate in the formation and regulation of liver inflammation and fibrosis. BMSCs secrete various soluble mediators, inhibit the proliferation and activation of various immune cells, and ultimately inhibit the activation of hepatic stellate cells. Research by Guo et al. found that MSCs transplantation in patients with liver cirrhosis can inhibit the proliferation of peripheral blood T lymphocytes, upregulate the expression of CD4, CD25, and CD127, regulatory T cells (Treg), and also alleviate the Treg/Th17 imbalance [16]. Liu et al. [17] also found that, during liver fibrosis, CD4+, CD25+, FoxD3, and other regulatory T cells in the liver increased, while NK cells decreased. CD4+, CD25+, and Foxp3+ cells are recognized regulatory T cell subsets in recent years, and they are the maintainers of peripheral immune tolerance. They can also inhibit or release IL-10, TGF-*β*, and other cytokines through cell-to-cell contact to inhibit the effect of T cells, which prevents the body from getting out of control. Xin et al. [18] found that BMSCs can inhibit the activation of T lymphocytes and inhibit the immune response by reducing the release of the inflammatory factor TNF-*β*. In addition, BMSCs can downregulate immune response and produce immune tolerance through autocrine and inducing T regulatory cells to produce and secrete immunosuppressive factors IL-10 and TGF-*β*. Macrophages play a central role in liver fibrosis and fibrosis regression [19]. In the process of liver fibre formation, pro-inflammatory M1 macrophages located near activated liver myofibroblasts secrete pro-fibrotic factors. This secretion promotes the increase of myofibroblast fibrogenesis, activation, proliferation, and chemotaxis. However, studies [20] have shown that MSCs can induce changes in cytokine-activated macrophages and promote fibrosis resolution.

### 2.3. Promoting the Degradation of Collagen Fibres

There are kinds of MMPs in the liver, of which the main function is to degrade extracellular matrix. The activation of MMPs is related to TIMPs. Under normal circumstances, metalloproteinases in hepatocyte matrix can degrade collagen fibres in hepatocyte matrix. When liver cirrhosis occurs, the TIMP secreted by activated HSCs increases and inhibits the activity of MMP, leading to a large increase of collagen fibres in liver tissue [21]. Khalifa et al. [22] used superparamagnetic nanometre iron oxide (SPIO) to label BMSCs and found that BMSCs can promote the regression of fibrous tissue in liver fibrosis. MMP-1 can degrade type I and III collagen, which is the key collagen in irreversible scars of liver cirrhosis. Matrix metalloproteinase-9 (MMP-9) specifically degrades type III collagen and gelatin to control the progression of liver fibrosis [23]. Du et al. [24] have studied the therapeutic effect of BMSCs transfected with human matrix metalloproteinase-1 (MMP-1) on carbon tetrachloride-induced hepatic fibrosis in rats. They suggested that BMSCs may be a potential cell source in preventing liver fibrosis and MMP1 gene may enhance the anti-fibrotic effect of BMSCs. The in vitro studies of Tanimoto et al. [25] found that BMSCs inhibit the activation of HSCs by regulating the expression of humoral factors and increase MMP-9, which helps to inhibit activated HSCs and improve fibrosis.

### 2.4. Beneficial to Hepatocyte Regeneration

Related studies have found that, under specific culture conditions, BMSCs can change their phenotype and play a metabolic function similar to that of hepatocytes. Some studies have shown that administered BMSCs can migrate to the liver [26]. However, direct transplantation of MSCs is available, but the number of hepatocytes in vivo that differentiate from MSCs transplanted is very low. Liver cells derived from MSCs are less than 1% of total liver cells, so liver function regeneration is extremely limited [27], which is still a clinical obstacle to BMSCs transplantation. BMSCs after transplantation have low survival rate and short half-life in host tissues. More recently, a number of other findings have indicated that the beneficial effects of MSC transplants should be ascribed to the capacity of MSCs to regulate tissue homeostasis and the immune system via a plethora of cytokines, growth factors, and differentiation factors [6]. At present, some studies have found that more BMSCs can be transformed into hepatocytes by some methods. Li et al. [28] found that autologous BMSCs can differentiate into hepatocytes and promote the regeneration of residual liver after portal vein embolization in patients with liver cirrhosis. In vitro, BMSCs can be induced to differentiate into precocious hepatocyte-like cells (HLCs) in indirect co-culture system with primary hepatocytes [29]. The mechanism may be to improve the microenvironment by alleviating liver cirrhosis and upregulating the expression of vascular endothelial growth factor, hepatocyte growth factor, IL-10, and MMP-9. The regeneration of hepatocytes is closely related to the repair of liver injury. Hu et al. [30] found that overexpression of liver nuclear factor- (HNF-) 4*α* directly induced immortalized human BMSCs to form HLCs. Li et al. [31] found that ultrasound radiation can induce BMSCs to transform into hepatocytes. Tan et al. found that Galectin-3 can induce rat BMSCs to differentiate into HLCs, and the differentiation efficiency can be improved further when combining with HGF. Inhibition of HIPPO signal pathway can improve the induction efficiency [32]. These studies provide a new strategy for the clinical application of BMSCs transplantation in the future.

### 2.5. Effect of Traditional Chinese Medicine on BMSCs in the Treatment of Liver Cirrhosis

Traditional Chinese medicine has a potential targeting function because of its unique views on disease, meridian tropism of traditional Chinese medicine, and promoting the operation of qi, blood, and body fluid, especially in the treatment of liver cirrhosis by tonifying kidney and activating blood circulation and removing blood stasis. At present, there are more and more clinical studies on traditional Chinese medicine combined with stem cell transplantation, and there are more and more cases of traditional Chinese medicine to improve the success rate of transplantation and liver function. The mechanism and the best dose will be increasingly clear. Traditional Chinese medicine can directly repair liver injury from multiple angles or indirectly improve the recovery of liver function by promoting BMSCs function or regulating transplanted cell colonization and immune system, so traditional Chinese medicine combined with stem cell transplantation will have a broader development prospect in the treatment of liver diseases.

### 2.6. Promoting the Proliferation of BMSCs

The therapeutic effect of BMSCs transplantation is closely related to the number of successful survival cells after transplantation. A large number of animal experiments and cytological studies have found that traditional Chinese medicine and its drug-containing serum can promote the proliferation of stem cells and increase the number of surviving cells after cell transplantation, thus improving the therapeutic effect. Wang et al. [33] studied the effects of different concentrations of Ligustrazine on the proliferation and adhesion of BMSCs and found that Ligustrazine can promote the proliferation of BMSCs and that appropriate concentration of Ligustrazine can significantly improve the adhesion of BMSCs. Ligustrazine is an alkaloid isolated from the rhizome of chuanxiong, which provides a reliable basis for clinical application of chuanxiong to regulate the proliferation of BMSCs and improve the adhesion of BMSCs. Chen et al. [34] found that the effective concentration range is 0.01∼3.0 mg/mL tortoise plate extract, which can increase the proportion of BMSCs in the proliferative phase and promote the proliferation of BMSCs. Huang et al. [35] studied the effect of different concentrations of astragalus polysaccharides on the proliferation of BMSCs and found that 1-3 mg/mL astragalus polysaccharides could promote the proliferation of BMSCs, in which the upregulation of SCF mRNA and SCF protein content may be the mechanism of astragalus polysaccharides promoting the proliferation of BMSCs in vitro. Peng et al. [36] found that astragaloside can promote the proliferation of human BMSCs in vitro, and its mechanism may be related to its promotion for the expression of SCF, VEGF, and SDF-1 mRNA in human BMSCs, which provides a theoretical basis for astragalus membranaceus to play a role in BMSCs transplantation. Icaritin (ICT) is an active ingredient of the genus *Epimedium*, a traditional Chinese medicine, with the potential to enhance the proliferation of MSCs. Lu et al. [37]found out that MSCs increased the survival rate of acute liver failure rats and reduced liver damage. MSCs cocultured with ICT exerted a greater therapeutic effect than MSCs alone. Further, the HGF/c-Met pathway played a key role in the antiapoptotic activity of MSCs, which was associated with the optimized efficacy of ICT.

## 3. Improving BMSCs Differentiating into HLCs

Chinese Materia Medical can not only stimulate the proliferation of BMSCs and improve the survival rate of BMSCs, but also improve the success rate of MSCs differentiating into HLCs. The differentiation of MSCs has been seen in two main routes: (1) directly into hepatocytes; (2) induced by cytokines such as hepatocyte growth factor (HGF) that is secreted by MSCs and injured liver tissue. Curcumin is the main active ingredient extracted from turmeric, which has the function of promoting blood circulation, removing blood stasis, promoting qi, promoting menstruation, relieving pain, and so on, so it is widely used in the treatment of liver diseases. Chen et al. [38] found that 20 ng/mL curcumin can induce BMSCs to differentiate into HLCs. To make it safer and better, we can combine with FGF-4 so as to reduce the concentration of curcumin. Cordyceps polysaccharide is the main effective component of *Cordyceps sinensis*. Liu et al. [39] found that, 14 days after induction, alpha-fetoprotein, albumin, keratin 18, and glycogen staining were all positive in Cordyceps polysaccharide group. It is confirmed that Cordyceps polysaccharides can induce rat BMSCs to differentiate into HLCs in vitro. Liu et al. [39] studied the effects of Cordyceps polysaccharides combined with BMSCs transplantation on the expression of MMP-13 and TIMP-1 in cirrhotic rats and found that Cordyceps polysaccharides combined with MSCs transplantation could reduce the expression of TIMP-1 in liver tissue, further enhance the expression of MMP-13, significantly promote collagen degradation, and reduce collagen deposition. Ye and Wang [40] found that the combined treatment of rhubarb and MSCs can promote hepatocyte regeneration and repair after liver injury.

### 3.1. The Mechanism of Herbal Formulas in the Treatment of Liver Fibrosis

Yiguanjian decoction is a famous prescription for the treatment of liver disease, which comes from the “Continued Cases of Well-Known Physicians” (a collection of medical records in ancient China). It was founded by Yupu Wei, a famous doctor in the Qing Dynasty, and is composed of Coastal Glehnia Root, Radix Ophiopogonis, Angelica sinensis, Radix Rehmanniae, Chinese wolfberry, Toosendan, and other herbs. It has been proved to reduce liver fibrosis. Fu et al. [41] found that Yiguanjian decoctions drug-containing serum could facilitate the differentiation of murine BMSCs into hepatocytes in vitro and has a synergistic effect with SDF-1 and HGF. By exploring the related mechanism of inducing BMSCs to differentiate into hepatocytes, Ping et al. [42] found that Yiguanjian decoctions serum can increase the expression of Alb, AFP, and HNF4a mRNAs of BMSC and promote the expression of CK18 protein. It is proved that Yiguanjian decoctions can induce BMSCs to differentiate into hepatocytes which has the function of mature hepatocytes. The mechanism may be related to the downregulation of Wnt/*β*-catenin signal pathway. Because of the therapeutic effect of this decoction on liver cirrhosis, the study of its mechanism in the treatment of liver cirrhosis is helpful to better apply it in the treatment of liver diseases. Chen et al. [43] found that fuzhenghuoxue formula enhanced hepatocyte differentiation from human embryonic stem cells. These enhancements by fuzhenghuoxue formulas are mediated through activation of canonical Wnt pathways and ERK pathways and inhibition of Notch pathway. Li et al. [44] studied the effect of zuoguiwan drug-containing serum on the differentiation into hepatocytes. They believe that kidney-tonifying prescriptions can promote the transformation of BMSCs into hepatocytes, regulate systemic function, and improve liver microenvironment. Wen et al. [45] explored the mechanism of biejiajianwan intervention on BMSCs through Notch signal pathway. It was found that biejiajianwan can promote the differentiation of BMSCs into HLCs, and its mechanism may be related to the regulation of Notch-1 protein and Notch-1mRNA expression in BMSCs Notch signal pathway. Wen et al. [45] believe that biejiajianwan combined with bone marrow stem cell transplantation can improve the effect of treatment of liver fibrosis, and it may work through the action of SDF-1/CXCR4 axis.

### 3.2. Problems and Prospect of BMSCs Transplantation in the Treatment of Liver Cirrhosis

Autologous BMSCs transplantation may be the best choice for cell therapy in patients with liver cirrhosis [46]. However, the uncertainty is how to make BMSCs play a successful and effective role in clinical application. Scholars have made relevant clinical studies on the role of BMSCs in the treatment of liver cirrhosis. It is still difficult to distinguish how many hepatocytes in patients are regenerated by BMSCs [29]. In spite of this, there are still many reports about the successful clinical application of BMSCs. There are several noteworthy areas in clinical trials that need to be further addressed, such as the best cell type, the best timing of treatment, the most effective cell count, and the best number of injections [47]. Besides, the survival time of transplanted cells is also important and we need to achieve a sustained effect. This requires immunohistochemical analysis of human-specific markers to observe these cells [48]. However, for clinical use, more complex techniques will need to be developed. For example, transplanted cells can be labelled so that they can be tracked. So, it is necessary to have advanced imaging techniques. These require constant exploration. In addition to the clinical efficacy of BMSCs in the treatment of liver cirrhosis, there are other problems that need to be solved urgently. Some studies have pointed out that MSCs may promote tumour growth in vivo, so people question the long-term effectiveness and potential carcinogenic risk of MSCs therapy. On the other hand, some studies have suggested that MSCs may have anti-tumour effect, which is related to the regulation of inflammatory environment of many tumours. MSCs can also interact with cancer cells and inhibit signal pathways related to tumour growth and cell division, so the safety of BMSCs transplantation also needs to be monitored. At the same time, the isolation, culture, and identification of BMSCs in vitro need to be further improved, so that they can better differentiate and expand into hepatocytes. About the indications and contraindications of direct transplantation and transplantation after vitro differentiation of BMSCs, and how to avoid vascular embolism in cell transplantation, these problems need to be further observed, solved, and improved in the treatment of liver cirrhosis with BMSCs. At present, how to use BMSCs safely and effectively in the treatment of end-stage liver disease is a key research field of many scholars. For various ways of using BMSCs in the treatment of liver cirrhosis, the research of traditional Chinese medicine provides some new clues. As we all know, traditional Chinese herbs monomer and traditional Chinese medicine prescription have the characteristics of “multi-component, multi-target, multi-channel, multi effect.” Therefore, to explore the target of traditional Chinese medicine intervention on BMSCs in the treatment of liver cirrhosis is helpful to focus on effective prevention and treatment of traditional Chinese medicine or prescription and provide scientific theoretical basis for clinical treatment of liver fibrosis and reversal of liver cirrhosis. However, the composition of traditional Chinese medicine is complex, and its specific mechanism of action and target still need to be further explored. Some traditional Chinese medicine prescriptions even contain hepatotoxic drugs. How to control the dosage in the process of use and how to reduce its toxic and side effects are also issues that need further study.

## 4. Conclusions

BMSC therapy is a promising cell therapy, which can treat liver fibrosis and reverse liver cirrhosis through a variety of ways, and has a certain therapeutic effect. In the subsequent experiment, how to modify BMSCs with safety and stability, as well as how to induce those cells to play a greater role in the treatment of liver cirrhosis, is a problem that still needs to be solved. Traditional Chinese medicine has its unique advantages in controlling the progression of liver cirrhosis and relieving clinical symptoms. The research on the intervention of MSCs in the treatment of liver cirrhosis by traditional Chinese medicine has received great attention and achieved many achievements. It is necessary to systematically sort out the theoretical basis of traditional Chinese medicine in the intervention of liver cirrhosis and MSCs to better expand the research ideas. We should also deeply excavate the effective components of traditional Chinese medicine, properly use the achievements of modern science and technology, and increase the research on the MSCs regulated by traditional Chinese medicine, in order to improve the ability of traditional Chinese medicine in liver diseases. It is believed that, with the further deepening of relevant research, traditional Chinese medicine will play a key role in the research and application of stem cells, which is very attractive for the development of effective anti-fibrosis therapy. Therefore, the study of BMSCs in the treatment of liver cirrhosis needs to be more in-depth and do a good job in basic research, so as to contribute to improving the therapeutic effect and quality of life of patients with liver disease.

## Data Availability

The data used to support the findings of this study are available from the corresponding author upon request.
